# Multiscale neural gradients reflect transdiagnostic effects of major psychiatric conditions on cortical morphology

**DOI:** 10.1038/s42003-022-03963-z

**Published:** 2022-09-27

**Authors:** Bo-yong Park, Valeria Kebets, Sara Larivière, Meike D. Hettwer, Casey Paquola, Daan van Rooij, Jan Buitelaar, Barbara Franke, Martine Hoogman, Lianne Schmaal, Dick J. Veltman, Odile A. van den Heuvel, Dan J. Stein, Ole A. Andreassen, Christopher R. K. Ching, Jessica A. Turner, Theo G. M. van Erp, Alan C. Evans, Alain Dagher, Sophia I. Thomopoulos, Paul M. Thompson, Sofie L. Valk, Matthias Kirschner, Boris C. Bernhardt

**Affiliations:** 1 McConnell Brain Imaging Centre, Montreal Neurological Institute and Hospital, McGill University, Montreal, QC Canada; 2 Department of Data Science, Inha University, Incheon, Republic of Korea; 3 Center for Neuroscience Imaging Research, Institute for Basic Science, Suwon, Republic of Korea; 4 Forschungszentrum Jülich, Jülich, Germany; 5 Max Planck Institute for Cognitive and Brain Sciences, Leipzig, Germany; 6 Max Planck School of Cognition, Leipzig, Germany; 7 Institute of Systems Neuroscience, Heinrich Heine University Düsseldorf, Düsseldorf, Germany; 8 Institute of Neuroscience and Medicine (INM-1), Forschungszentrum Jülich, Jülich, Germany; 9 Center for Cognitive Neuroimaging, Donders Institute for Brain, Cognition and Behavior, Radboud University, Nijmegen, The Netherlands; 10 Department of Psychiatry, University of Groningen, University Medical Center Groningen, Groningen, The Netherlands; 11 Donders Institute for Brain, Cognition and Behaviour, Radboud University, Nijmegen, The Netherlands; 12 Department of Human Genetics and Department of Psychiatry, Radboud university medical center, Nijmegen, The Netherlands; 13 Centre for Youth Mental Health, The University of Melbourne, Melbourne, VIC Australia; 14 Orygen, Parkville, VIC Australia; 15 Departments of Psychiatry and Anatomy and Neuroscience, Amsterdam University Medical Centers, Vrije Universiteit Amsterdam, Amsterdam, The Netherlands; 16 SA Medical Research Council Unit on Risk & Resilience in Mental Disorders, Dept of Psychiatry and Mental Health, University of Cape Town, Cape Town, South Africa; 17 NORMENT Centre, Division of Mental Health and Addiction, Oslo University Hospital &Institute of Clinical Medicine, University of Oslo, Oslo, Norway; 18 KG Jebsen Centre for Neurodevelopment, Institute of Clinical Medicine, University of Oslo, Oslo, Norway; 19 Imaging Genetics Center, Mark and Mary Stevens Neuroimaging and Informatics Institute, Keck School of Medicine, University of Southern California, Los Angeles, CA USA; 20 Department of Psychology and Behavioral Health, The Ohio State University, Columbus, OH USA; 21 Tri-institutional Center for Translational Research in Neuroimaging and Data Science (TReNDS), Georgia State University, Georgia Institute of Technology and Emory University, Atlanta, GA USA; 22 Neuroscience Institute, Georgia State University, Atlanta, GA USA; 23 Clinical Translational Neuroscience Laboratory, Department of Psychiatry and Human Behavior, University of California Irvine, Irvine, CA USA; 24 Center for the Neurobiology of Learning and Memory, University of California Irvine, Irvine, CA USA; 25 Division of Adult Psychiatry, Department of Psychiatry, Geneva University Hospitals, Geneva, Switzerland

**Keywords:** Neuroscience, Psychiatric disorders

## Abstract

It is increasingly recognized that multiple psychiatric conditions are underpinned by shared neural pathways, affecting similar brain systems. Here, we carried out a multiscale neural contextualization of shared alterations of cortical morphology across six major psychiatric conditions (autism spectrum disorder, attention deficit/hyperactivity disorder, major depression disorder, obsessive-compulsive disorder, bipolar disorder, and schizophrenia). Our framework cross-referenced shared morphological anomalies with respect to cortical myeloarchitecture and cytoarchitecture, as well as connectome and neurotransmitter organization. Pooling disease-related effects on MRI-based cortical thickness measures across six ENIGMA working groups, including a total of 28,546 participants (12,876 patients and 15,670 controls), we identified a cortex-wide dimension of morphological changes that described a sensory-fugal pattern, with paralimbic regions showing the most consistent alterations across conditions. The shared disease dimension was closely related to cortical gradients of microstructure as well as neurotransmitter axes, specifically cortex-wide variations in serotonin and dopamine. Multiple sensitivity analyses confirmed robustness with respect to slight variations in analytical choices. Our findings embed shared effects of common psychiatric conditions on brain structure in multiple scales of brain organization, and may provide insights into neural mechanisms of transdiagnostic vulnerability.

## Abstract (web summary)

Individuals across six major psychiatric conditions from the ENIGMA consortium reveals a shared morphological effect, following a sensory-fugal axis, which is related to microstructural gradient and neurotransmitter axes.

## Introduction

Mental illness refers to a wide range of psychiatric conditions affecting individuals, families, and health systems at large^1^. While conventional psychiatric nosology classifies mental illness into distinct categories mainly based on descriptive symptoms and behaviors^2^, high co-occurrence of symptoms across disorders as well as transdiagnostic risk factors have prompted reconceptualization of mental illnesses along symptom dimensions^3–8^. Investigation of transdiagnostic effects may, thus, benefit detailed characterization of shared alterations across different psychiatric conditions and may identify direct brain-behavior associations that capture multiple symptom classes and mask clinical heterogeneity.

The shared components across major psychiatric diagnosis may be more clearly distinguishable at the neural level^4,9^, as behavioral characterization likely involves complex interactions with society and the environment^10^. Structural magnetic resonance imaging (MRI), in particular, offers high spatial precision to help resolve the pattern of shared transdiagnostic effects across the cortical surface^4,11–16^. Prior case-control studies have reported reproducible patterns of structural alterations in cohorts with psychiatric diagnoses relative to controls^17–21^, often pointing to widespread changes in cortical morphology in these conditions. More recently, efforts have been expanded to a transdiagnostic perspective, aiming to identify structural compromise that is shared across different diagnoses^22–24^. To ensure the sensitivity of such efforts and to strengthen reproducibility, it becomes increasingly relevant to pool these investigations across multiple sites. One such initiative, spearheaded by the Enhancing NeuroImaging Genetics through Meta-Analysis (ENIGMA) consortium, has aggregated MRI and phenotypic data in thousands of healthy individuals and those with a psychiatric diagnosis^25^. Moreover, dedicated ENIGMA working groups have confirmed neuroanatomical disruptions in major psychiatric indications, including autism spectrum disorder (ASD)^26^, attention deficit hyperactivity disorder (ADHD)^27^, major depressive disorder (MDD)^28^, obsessive-compulsive disorder (OCD)^29^, bipolar disorder (BD)^30^, and schizophrenia (SCZ)^31^, pointing to widespread changes in cortical morphology in each of these different conditions. Also fostered by the open dissemination of condition-related effect size maps through the primary ENIGMA papers and their aggregation within the recently developed ENIGMA toolbox^32^, it has now become possible to systematically study these effects.

In addition to providing robust evidence of neuroanatomical signatures associated with each of these conditions, an emerging body of studies has pooled data across different indications to identify shared anomalies of psychiatric conditions^33,34^. In an effort to identify factors contributing to the topography of cross-disorder brain changes, a recent study has taken this approach one step further and examined associations to postmortem gene expression data, searching for spatially co-varying gene lists that may carry susceptibility to transdiagnostic disease effects. This study revealed that transdiagnostic effects may generally be more marked in regions with greater expression of CA1 pyramidal genes that were suggested to play a role in regulating cortical thickness. Beyond these molecular risk factors, a broad range of cellular, metabolic, and functional properties of brain regions may contribute to regional susceptibility, but such an association remains underexplored. An influential theory, also referred to as the “structural model”, posits that the internal microstructural and connectional markup of different brain regions, in particular their laminar differentiation and cortico-cortical connectivity patterns, may represent mesoscale features associated with the potential of a region to show plasticity, and to be susceptible to pathological processes^35^. According to this framework, paralimbic cortices with low laminar differentiation and associated connectivity profiles may be more susceptible to effects of neurological as well as psychiatric disorders. Here, we tested this approach by aligning transdiagnostic effects with maps of microstructural variations derived from both in vivo imaging and 3D postmortem histology^36–39^. In recent work, the application of nonlinear eigenvector decomposition techniques to imaging and histology datasets identified a sensory-fugal gradient that radiates from sensory and motor areas with strong laminar differentiation and higher myelination towards heteromodal association and paralimbic regions with less clear lamination and lower myelin content. Of note, similar yet not completely corresponding gradients have also been derived from the analysis of intrinsic functional connectivity patterns obtained from resting-state functional MRI^38–40^. In line with foundational neuroanatomical conceptualization^35,41,42^, an emerging literature has underscored a correspondence between such data-driven sensory-fugal gradients and region-to-region variations in cortical plasticity and genetic control^40,43–47^, suggesting that these likely help understand susceptibility to common brain disorders as well^40,43,48–52^.

Examining associations between shared morphological alterations and receptor architecture may provide additional opportunities for the contextualization of transdiagnostic effects. In the healthy brain, neurotransmitter systems are indeed broadly implicated in region-to-region variations of synaptic plasticity, neural dynamics, and inter-network communication. Moreover, recent initiatives have aggregated maps outlining the spatial distributions of different neurotransmitter systems in vivo, based on positron emission tomography (PET) and single photon computed emission tomography (SPECT) studies sensitive to different receptor and transporter types^53–59^. Such mapping can complement microstructural and functional connectivity contextualization of transdiagnostic findings, promising insights into additional molecular factors contributing to regional susceptibility. Beyond the mapping of regional variations in the receptor architecture of the neurotypical brain, neurotransmitter imbalances have been described in several psychiatric conditions. Work in SCZ and depression, for example, implicated a role of dopamine and serotonin^60–63^, and more recent work in BD and SCZ demonstrated associations between neurotransmitter and functional network imbalances^64^.

Here, we studied the association between multiscale neural organization and transdiagnostic effects on cortical morphology across six major psychiatric conditions (ASD, ADHD, MDD, OCD, BD, and SCZ). Aggregating data from thousands of patients and healthy controls previously studied across several ENIGMA working groups^26–31^, we defined the shared effect using principal component analysis. The robustness of the shared effect was further cross-validated based on openly aggregated effect size maps from the ENIGMA toolbox^32^. The shared dimension was contextualized across multiple neural scales. This involved systematic assessment of spatial associations to (i) in vivo myeloarchitecture and intrinsic functional connectivity, (ii) postmortem 3D cytoarchitecture, and (iii) in vivo maps of neurotransmitter distributions. Notably, in addition to assessing specific associations, we also adopted supervised machine learning to identify joint spatial associations between the above neural features and the common dimension of morphological alterations. Multiple sensitivity analyses verified robustness of our findings.

## Results

### Study overview and participants

We obtained case-control maps of cortical thickness differences in patients relative to controls, resulting from several ENIGMA working groups aggregated by a previous study that included a total of 28,546 participants across 145 independent cohorts (1821 ASD, 1815 ADHD, 2695 MDD, 2274 OCD, 1555 BD, 2716 SCZ; 15,670 site-matched controls Supplementary Table 1)^33^. We then associated principal dimensions of morphological with (i) in vivo myeloarchitecture and functional connectivity gradients obtained from the Human Connectome Project (HCP)^65^, (ii) postmortem cytoarchitecture, by cross-referencing data to a ultra-high resolution 3D histological human brain model^66^, and (iii) in vivo neurotransmitter topographies provided by PET/SPECT studies^53–59^. Approaches are openly available and replicable via the ENIGMA toolbox (https://enigma-toolbox.readthedocs.io)^32^. See Methods for more details.

### Shared dimensions of structural alterations across psychiatric conditions

**Fig. 1:**
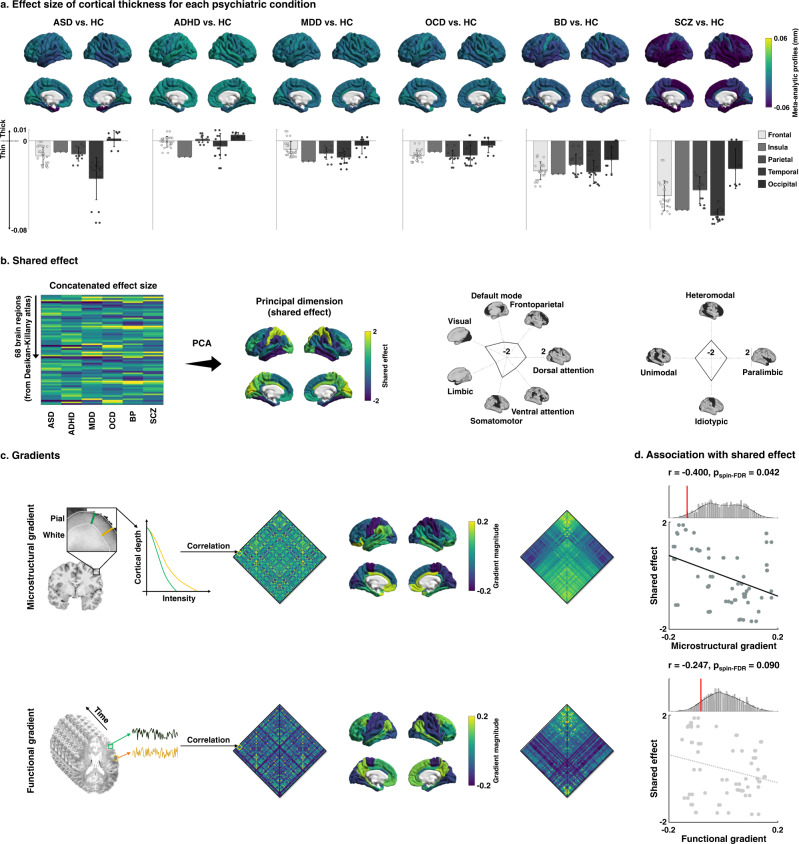
Shared disease effect and associations to connectivity gradients. **a** Meta-analytic profiles of cortical thickness differences (unit in mm) in patients with each psychiatric condition relative to matched controls. Positive/negative values indicate increases/decreases in cortical thickness in patients relative to controls. Mean values of the regions involved in the same cortical lobes with SD are reported with bar plots. **b** The shared effect was identified through principal component analysis (PCA) applied to the concatenated effect size map. Spider/Radar plots stratify the effects according to functional communities^68^ and cortical hierarchy levels^41^. **c** The microstructural and functional connectivity gradients were generated by applying nonlinear dimensionality reduction techniques to the group averaged connectivity matrix (middle left), and each connectivity matrix was reordered (right) according to the first gradients (middle right). **d** Spatial correlations of each gradient with the shared effect map are shown in the scatter plots. The distribution of correlation coefficients across 1,000 spin-tests are reported with histograms, and the actual *r*-values are represented with red bars. ASD autism spectrum disorder, ADHD attention deficit hyperactivity disorder, MDD major depressive disorder, OCD obsessive-compulsive disorder, BD bipolar disorder, SCZ schizophrenia, HC healthy controls, spin-FDR spin-test followed by false discovery rate.

Following standardized ENIGMA protocols (http://enigma.ini.usc.edu/protocols/imaging-protocols/), gray matter thickness for 68 cortical regions of the Desikan–Killiany atlas^67^ was calculated, and meta-analytic between-group differences in cortical thickness were assessed using inverse variance-weighted random-effects models (Fig. 1a)^33^. Using principal component analysis adopted in a recent study^33^, we estimated the shared disease dimensions explaining structural alterations across six conditions (Fig. 1b). The first dimension/component explained 55.7% of variance, and differentiated sensory/motor systems having positive scores from transmodal/paralimbic areas with negative scores (for details, and information on the other dimensions/components, see Supplementary Fig. 1a). Stratifying the first dimension according to intrinsic functional communities^68^, it indeed differentiated somatomotor/visual from default/frontoparietal/limbic networks (Fig. 1b). Similar spatial patterns were observed across an atlas of the putative primate cortical hierarchy^41^, differentiating idiotypic/unimodal from heteromodal/paralimbic levels.

Several sensitivity analyses confirmed and further expanded these findings. Firstly, scores on the principal dimension translated into mean effect size of morphological alterations across case-control analyses, with paralimbic regions showing the strongest atrophy in patients relative to controls, while sensory/motor regions showed the least gray matter alterations (Supplementary Fig. 1b). Compared to the dimensional analysis that highlighted paralimbic as well as sensory/motor regions with opposite ends, this analysis confirmed that paralimbic regions are most vulnerable to the impact of neuropsychiatric conditions, and primary motor cortex is least vulnerable. Secondly, we directly ran principal component analysis on previously reported effect size maps (Cohen’s d) concatenated across disorders, sourced from the ENIGMA toolbox^32^ (Supplementary Fig. 1c). The first principal dimension was highly similar to ours (*r* = 0.552, spin-test *p*_spin_ < 0.001), suggesting robustness. Thirdly, the shared disease effect resembled the effects of each condition, with the strongest spatial similarity to SCZ and BD, followed by MDD, ADHD, ASD, and OCD (spin-test followed by false discovery rate (FDR) correction, *p*_spin-FDR_ < 0.001; Supplementary Fig. 2), indicating that the shared effect captured structural alterations from each condition. Fourthly, we re-evaluated the shared dimension using leave-one-condition-out procedure (see Methods), and observed largely consistent results with the shared effect based on all conditions (*r* > 0.9, *p*_spin-FDR_ < 0.001; Supplementary Fig. 3), indicating that a single condition with strong meta-analytic profile did not determine the shared disease effect. Finally, we associated three alternative shared disease maps (including principal dimension (see Fig. 1b), mean effect (see Supplementary Fig. 1b) of meta-analytic profiles, and principal dimension of Cohen’s d map (see Supplementary Fig. 1c)) with previously published maps of cortical expansion and functional reconfiguration^69^ to examine whether the shared disease dimension reflects evolutionary expansion. We found low-to-moderate correlations (*r* = −0.256, *p*_spin_ = 0.036 for shared disease dimension; *r* = −0.193, *p*_spin_ = 0.087 for mean effect size; *r* = -0.480, *p*_spin_ < 0.001 for principal component of Cohen’s d maps).

### Associations with cortical myeloarchitecture and functional connectivity gradients

To assess in vivo micro- and macroscopic properties of the shared disease dimension on cortical morphology, we first examined its spatial association with myeloarchitecture and intrinsic functional connectivity gradients^38,40^ (see Methods; Fig. 1c). The microstructural gradient was derived from inter-regional similarity matrices of intracortical profiles of myelin-sensitive MRI^38^, and runs from sensory/motor regions with high laminar differentiation and high intracortical myelin content towards paralimbic cortices with reduced laminar differentiation and low myelin content^38^. The intrinsic functional gradient was derived from resting-state functional MRI connectivity. While it also runs from sensory/motor to transmodal areas, it finds its apex in the heteromodal default mode and frontoparietal networks, and not in paralimbic cortices^40^. Associating the patterns of shared dimension with these two in vivo gradients, we observed a negative association with the microstructural gradient (*r* = −0.400, *p*_spin-FDR_ = 0.042) and a negative trend with the functional connectivity gradient (*r* = −0.247, *p*_spin-FDR_ = 0.090; Fig. 1c). In other words, transdiagnostic morphological alterations follow sensory-fugal gradients of cortical organization, in particular, the microstructural gradient that differentiates sensory/motor areas with high myelination and distinct lamination from paralimbic areas with low myelin content and reduced laminar differentiation.

### Cytoarchitectonic associations

**Fig. 2:**
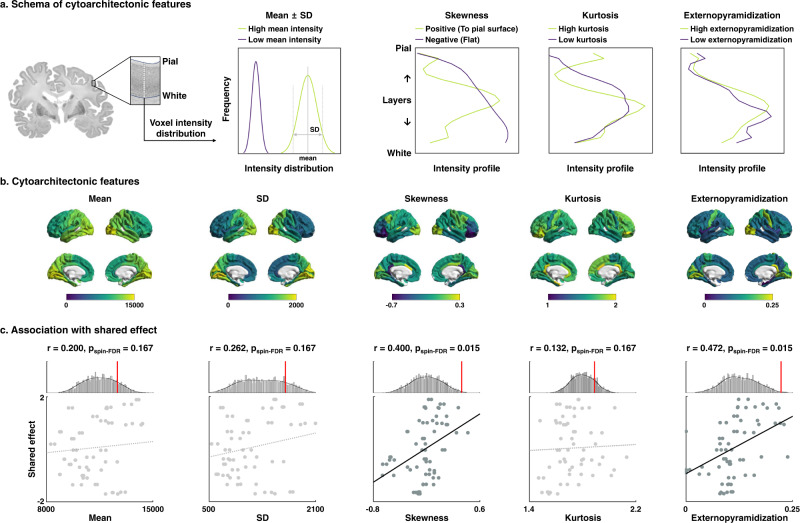
Cytoarchitectonic associations with the shared disease effect. **a** Cytoarchitectonic moment features of mean, SD, skewness, and kurtosis, as well as externopyramidization of intracortical intensity profile were calculated from the postmortem human brain, and **b** plotted on brain surfaces. **c** Spatial correlations between the features and shared effects are shown on scatter plots. The distributions of correlation coefficients across 1000 spin-tests are reported with histograms, and the actual *r*-values are represented with red bars. SD standard deviation, spin-FDR spin-test followed by false discovery rate.

We furthermore examined associations of the shared disease effect with inter-regional variations in cortical cytoarchitecture^37^, using BigBrain, a 3D histological reconstruction of a postmortem human brain^66,70^. We calculated cortex-wide variations in cytoarchitecture using two alternative approaches. First, we obtained intracortical intensity profiles and calculated their statistical moments, i.e., mean, SD, skewness, and kurtosis (Fig. 2a, b). In both classic cytoarchitecture analysis and more recent work, these features have been shown to relate to inter-areal microstructural differentiation^39,71^. In particular, the skewness moment describes a robust spatial transition from areas with low laminar differentiation and negative skewness to those with high laminar differentiation and positive skewness^71–73^. Moreover, we computed externopyramidization^74^, describing a gradual shift of intensity profiles across cortical layers that has been suggested to also differentiate areas on the lower end of the cortical hierarchy from those that are higher up due to hierarchical shifts in laminar projection profiles^75^ (Fig. 2a, b). Notably, while both skewness and externopyramidization describe overall sensory-fugal patterns, they do so in complementary ways (*r* = 0.015, *p*_spin_ = 0.506), with skewness differentiating mainly prefrontal and posterior cingulate regions from visual, auditory, and frontocentral regions while externopyramidization clearly differentiates postcentral and visual regions from the rest of the brain. Importantly, however, spatial correlations between these features and the principal disease dimension indicated relations to both features (skewness: *r* = 0.400, *p*_spin-FDR_ = 0.015; externopyramidization: *r* = 0.472, *p*_spin-FDR_ = 0.015; Fig. 2c). In other words, transdiagnostic cortical thickness decreases were more likely in paralimbic regions with low skewness and low externopyramidization, independently confirming that those areas with low laminar differentiation were more likely to show transdiagnostic cortical alterations. In addition to the associations with BigBrain cytoarchitectural features, we additionally examined associations of the shared disease effect with intracortical profile moments and externopyramidization calculated from in vivo myelin-sensitive MRI i.e., T1w/T2w measures obtained from the HCP database. We observed largely consistent results (Supplementary Fig. 4), suggesting robustness.

### Associations with distributions of neurotransmitter systems

**Fig. 3:**
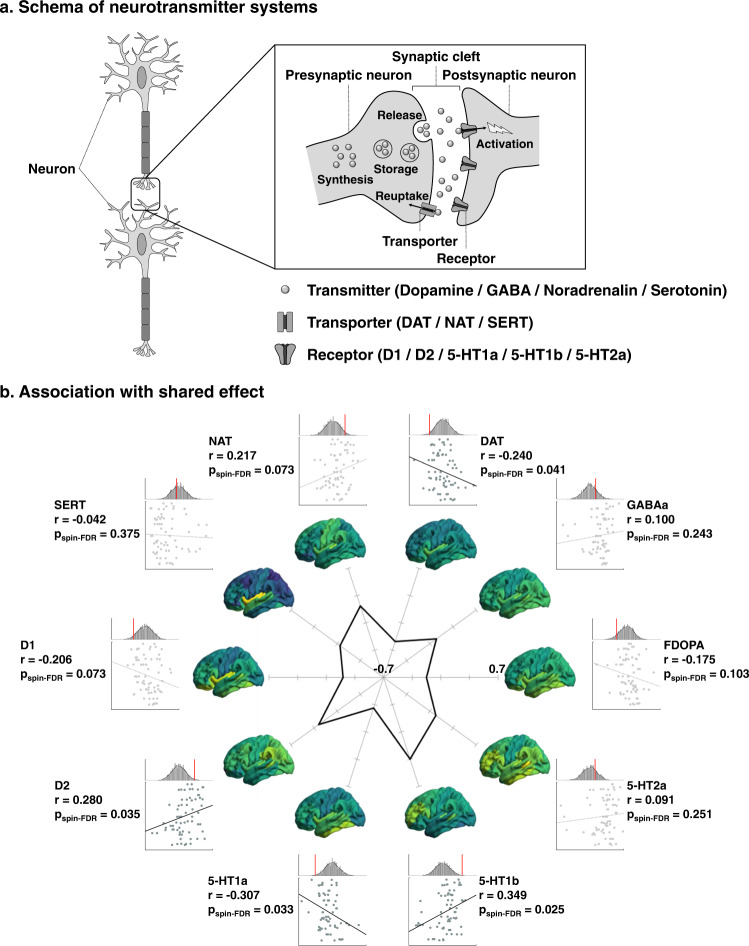
Associations of neurotransmitter systems with shared disease effect. **a** Schema of neurotransmitter systems of transmitters, transporters, and receptors. **b** Spatial correlations of each neurotransmitter map with shared effect are shown on scatter plots. The distributions of correlation coefficients across 1000 spin-tests are reported with histograms, and actual *r*-values are reported with red bars. The spider plot shows correlation coefficients. Cortex-wide spatial maps of the transmitter systems are reported on brain surfaces. FDOPA 18 F fluorodopa, DAT dopamine transporter, NAT noradrenaline transporter, SERT serotonin transporter, spin-FDR spin-test followed by false discovery rate.

Neurotransmitter contextualization leveraged JuSpace^53^, an open access toolbox that disseminates in vivo PET/SPECT data sensitive to ten different transmitters/transporters/receptors from independent studies in healthy human adults^54–59^ (Fig. 3a). Associating the shared dimension with cortex-wide neurotransmitter maps, we observed positive associations with D2 and 5-HT1b receptor densities (D2: *r* = 0.280, *p*_spin-FDR_ = 0.035; 5-HT1b: *r* = 0.349, *p*_spin-FDR_ = 0.025), and negative correlations with dopamine transporter and 5-HT1a receptor density (DAT: *r* = −0.240, *p*_spin-FDR_ = 0.041; 5-HT1a: *r* = −0.307, *p*_spin-FDR_ = 0.033; Fig. 3b). The results indicate that common cortical alteration patterns across psychiatric and neurodevelopmental conditions may be reflected by serotonergic and dopaminergic systems. More specifically, higher transdiagnostic cortical atrophy was related to higher 5-HT1a and lower 5-HT1b, as well as higher DAT and lower D2 receptor density.

### Associations of multiscale features with other shared dimensions

We investigated associations of other principal components of cortical morphological alterations (Supplementary Fig. 1a), instead of first principal component, to multiscale neural features. We found that the first dimension showed significant associations to cytoarchitecture/microstructure features, while the second dimension showed higher (and inverted) associations to the functional gradient (Supplementary Table 2). The results indicate that the first principal dimension represents a more sensory-paralimbic axis, and the second dimension is a more sensory-heteromodal axis. In addition to cortical thickness, we investigated a shared disease dimension based on the surface area. The dimension showed a somatomotor-visual/frontoparietal pattern (Supplementary Fig. 5), which was different from the shared effect based on cortical thickness (*r* = −0.030, *p*_spin-FDR_ = 0.446). The surface area-based shared dimension was significantly associated with SERT (*r* = 0.274, *p*_spin-FDR_ = 0.029), 5-HT2a (*r* = −0.297, *p*_spin-FDR_ = 0.011), and GABAa (*r* = −0.325, *p*_spin-FDR_ = 0.005; Supplementary Table 3), suggesting higher sensitivity to serotonergic and GABAergic systems.

### Machine learning prediction of the shared disease effect

**Fig. 4:**
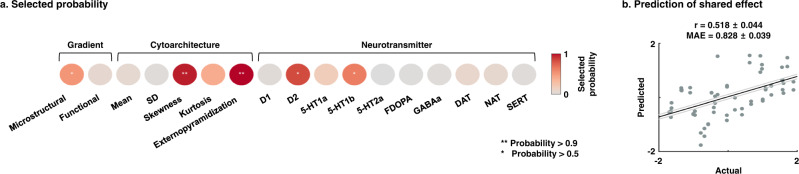
Association between the shared disease effect and multiscale features using machine learning. **a** Probability of the selected features across five-fold nested cross-validations and 100 repetitions for predicting the shared disease effect. The frequently selected features are reported with asterisks. **b** Linear correlation between actual and predicted values of the effects is shown on a scatter plot. The black line indicates mean correlation and the gray lines represent the 95% confidence interval for 100 iterations with different training/test datasets. SD standard deviation, FDOPA 18 F fluorodopa, DAT dopamine transporter, NAT noradrenaline transporter, SERT serotonin transporter, MAE mean absolute error.

As a final analysis, we used supervised machine learning to predict the first shared dimension using the above multiscale features. Specifically, we leveraged least absolute shrinkage and selection operator (LASSO) regression^76^ with five-fold nested cross-validation^77–80^ to predict the cross-condition effect using concatenated multiscale features (see Methods; Fig. 4a). Repeating the analysis for 100 times with different training and test dataset subsplits, we could reliably predict the spatial pattern of the shared disease dimension (mean ± SD, *r* = 0.518 ± 0.044, mean absolute error (MAE) = 0.828 ± 0.039, permutation-test *p*_perm_ < 0.001; Fig. 4b). Cytoarchitectural skewness and externopyramidization, followed by D2 and 5-HT1b receptors, as well as the microstructural gradient were frequently selected across cross-validations and repetitions (Fig. 4a). When considering each psychiatric condition separately, we could find significant prediction performances, but the features selected diverged across conditions (Supplementary Fig. 6).

## Discussion

The current work determined cortex-wide variations in susceptibility to morphological alterations across six major psychiatric conditions (i.e., ASD, ADHD, MDD, OCD, BD, and SCZ), and cross-referenced these spatial patterns against multiscale cortical organization. Specifically, complementing earlier case-control MRI studies performed separately in common neuropsychiatric conditions^17,26–31,81^, we applied dimensional decomposition to cortical morphological data. We identified a shared dimension that followed a sensory-paralimbic pattern of increasing susceptibility to morphological alterations in paralimbic regions, which was robust across different data and approaches, and consistent with prior work on shared functional imbalances^82,83^. Albeit prior functional connectivity-based findings highlighted heteromodal association and our structural morphological alterations highlighted paralimbic regions, the findings converge that higher order brain areas are vulnerable to multiple psychiatric conditions. Expanding from a prior study that reported transdiagnostic effects to be highest in brain regions expressing genes for pyramidal CA1 cells, pointing already to  potentially increased susceptibility of limbic allocortices^33^, here, we characterized the transdiagnostic effects across multiple scales of neural organization. Specifically, we contextualized the shared disease effect on MRI-derived morphology with respect to (i) in vivo MRI measures sensitive to cortical myeloarchitecture and intrinsic functional connectivity^38,40^, (ii) postmortem measures of cytoarchitecture, in particular laminar differentiation^37,39,66,71^, and (iii) in vivo PET/SPECT maps of cortical neurotransmitter systems^53–59^. Moreover, and in addition to studying specific associations between shared disease effects and individual features, we employed a supervised machine learning paradigm to synergistically assess the utility of multiscale neural features in explaining the shared disease effect. Our findings emphasize that microstructurally determined gradients, differentiating sensory/motor and paralimbic cortices^38,40,84^ can help to compactly describe cortex-wide susceptibility to transdiagnostic effects of common mental health conditions. Overall heightened susceptibility was furthermore associated with two neurotransmitter markers, serotonin, and dopamine. Altogether, our work extended prior work in terms of (i) demonstrating robustness of a shared disease dimension across psychiatric conditions via multiple sensitivity analyses, (ii) providing a framework that integrates multiscale neural organization with the transdiagnostic disease effect on cortical morphology, and (iii) assessing the synergistic value of different cortical features to explain the shared effect, which may advance our understanding of neuropathology in psychiatry, and may inform the development of diagnostic and treatment strategies that cut across traditional disease boundaries.

Whether our findings also suggest a shared disease mechanism remains to be established. Prior histopathological assessments in people with ASD, MDD, OCD, BD, and SCZ have reported common cellular alterations, in particular, reduced neuronal and glial densities as well as neuronal size in different cortical areas^85–92^. Moreover, a growing literature has shown shared genetic risk factors across major psychiatric conditions^93^. Studies have implicated genes involved in several synaptic pathways^93^, for example, common gene variants in cell adhesion and glutamate receptor pathways in ASD and SZ^94–98^, as well as those in calcium signaling in BD and SZ^99^. Together, the studies suggest that shared cellular and molecular risk factors may  influence structural plasticity and lead increased disease susceptibility of psychiatric conditions^93^, providing the rationale of investigating multiscale neural properties. As the first analysis, we defined the in vivo microstructural cortical gradient using a recently-introduced procedure^38^, which identified axes of cortico-cortical differentiation based on the similarity of myelin-sensitive MRI profiles sampled across cortical depths. In healthy adults and adolescents^38,71^, this approach has revealed a robust sensory-fugal cortical gradient running from sensory/motor areas with marked laminar differentiation and high myelin content towards paralimbic cortices with low overall myelination and rather agranular cortical profiles. By showing an association between the shared dimension and this microstructural gradient, we demonstrated a heightened susceptibility of paralimbic cortices to disease-related cortical thickness changes. Several features of the paralimbic cortex may underscore its increased susceptibility. On the one hand, paralimbic architecture may permit an increased potential for brain plasticity. This includes a lower neuronal density in paralimbic regions compared to eulaminate cortices, as well as increased dendritic arborization and synaptic density^35^. Compared to other regions, paralimbic areas also continue to express developmental markers long into adulthood, such as growth-associated protein GAP-43^100^. Furthermore, paralimbic cortices have a protracted myelination and lower myelin content relative to sensory/motor areas. The role of intracortical myelination in plasticity is likely complex, but several streams of evidence point to the role of myelin acting as a buffer against plasticity. In addition to acting as an insulator for electrical transmission, myelin associated growth inhibitors limit activity and experience-induced axon sprouting, with downstream effects on synaptic plasticity^101^. Reduced myelin content, together with increased complexity of dendritic arborization in transmodal and paralimbic regions may render cortical microstructure in these regions more susceptible to pathological alterations, which would echo observations in other conditions. For example, the core pathological substrates of drug-resistant temporal epilepsy is thought to be localized in limbic/paralimbic regions^102–104^, and prior work has suggested rather specific changes in myelin and microstructural proxies in these areas^105,106^. Similar findings have been observed in neurodegenerative conditions such as Alzheimer’s disease^45,107,108^, where pathology spreads from disease epicenters in paralimbic allocortices to invade more widespread cortical/subcortical networks, but also depression^109^ and autism^110,111^. These findings collectively indicate that cellular and molecular features of paralimbic cortices and their cortico-cortical pathways promote brain plasticity as well as higher metabolic activity, and are, thus, likely more vulnerable to both developmental as well as acquired disruptions than other regions, supporting the hypothesis that their cortical type predisposes to a heightened vulnerability for an impact of neuropsychiatric conditions on alterations in brain morphology^35^.

Studying a postmortem 3D model of the human brain, BigBrain^66^, we obtained supporting confirmation for the above association between cortical microstructure and disease-related susceptibility. In particular, we discovered similarly marked associations between the shared disease dimension and laminar profile skewness as well as externopyramidization, two complementary features tapping into depth-dependent shifts in the distribution in cell densities^39,74^. In prior work, profile skewness, in particular, was found to discriminate unimodal granular cortices from agranular/dysgranular paralimbic regions at a cortex-wide level^71^, and accurately delineated the iso-to-allocortical axis in the mesiotemporal lobe system^72^. Studying typical adolescent development, changes in profile skewness of myelin-sensitive MRI contrasts have furthermore been reported to spatially co-localize with expression patterns of genes enriched in oligodendrocytes^71^. As a complementary feature of the laminar organization, externopyramidization indexes the ratio of neuronal densities between supragranular and infragranular cortical layers. It increases when the cortex is cytoarchitectonically more differentiated, which happens in primary areas with a marked layer 4^74^. Thus, the association of these cortical depth-dependent cytoarchitectural features with the shared disease effect confirms the in vivo findings with ultra-high-resolution cytoarchitecture data suggesting that paralimbic areas, sensitive to transdiagnostic cortical alterations, are less laminarly differentiated. Furthermore, prior cellular and transcriptomic studies indicate regional susceptibility of synaptic elements as well as mutated genes in schizophrenia^112,113^ and bipolar disorder^114^. Indeed, major depression may be associated with atrophy of neurons in limbic regions^115^, pointing histopathological susceptibility of paralimbic areas in psychiatric conditions.

We also observed a marginal association between the transdiagnostic effect on brain structure and the principal functional connectivity gradient, but findings were overall weaker than for the above in vivo and postmortem derived microstructural gradients. Despite an overall convergence between structure and function in showing sensory-fugal gradients, prior work noted some divergence between sensory-heteromodal functional connectivity gradients^40^ and sensory-paralimbic microstructure/cytoarchitecture gradients^38^. This is in particular notable with respect to the heteromodal *vs* paralimbic anchor that these two gradients radiate towards. Mounting evidence suggests that heteromodal systems, such as the default mode network, decouple from microstructurally defined axes of brain organization that mainly describe differences in laminar differentiation^38,116^. That work has also shown that regions with strong microstructure-function decoupling also host more flexible cognitive functions, and have marked cross-species differences between humans and nonhuman primates. Considering that difference, the current work shows an association between transdiagnostic disease effects and microstructural (i.e., sensory-paralimbic) gradients but not between transdiagnostic disease effects and functional (i.e., sensory-heteromodal) gradients. As such, the above divergence suggests increased specificity of the sensory-paralimbic gradient with respect to disease-related vulnerability, which is likely more determined by the microstructural context of cortical areas compared to their placement within cortical functional hierarchies. It is nevertheless important to underscore that vulnerability and susceptibility are likely affected by multiple factors and be reflected in different structural and functional gradient axes^48,117–120^. These considerations collectively motivate caution in interpreting the observed associations, and also likely rule out a single mechanism underlying the observed association between the studied gradients and transdiagnostic effects.

In addition to our findings showing overall associations between the transdiagnostic effect and sensory-fugal microstructural gradients, we observed associations to the spatial distribution of different neurotransmitter systems derived from in vivo neuroimaging. Notably, associations were seen both to serotonin (5-HT1a and 5-HT1b) and dopamine receptors and transporters (DAT/D1 and D2), two previously reported markers of mental health and targets for pharmacological treatments^64,121–128^. In both cases (i.e., 5-HT1a vs 5-HT1b, DAT/D1 vs D2), associations to the disease effect were of opposite polarity, confirming prior work in rodents^129–133^ and humans^134–137^. Associations with in vivo neurotransmitter topographies provide a way of indirectly assessing the relationship between shared alterations of cortical morphology and neurotransmitter systems so that we can understand putative mechanisms of shared morphological alterations, extending prior work in rodents and humans. As different tracers may have variable sensitivity/specificity across cortical regions and, and as PET data may suffer from relatively coarse spatial resolution and partial volume effects, future studies are required to expand these findings based on a broader array of tracers and based on potentially more detailed techniques, such as 3D receptor autoradiography^138,139^.

Our study has limitations. First, comorbidities and medication may contribute to the shared disease effect. Comorbidities are common in psychiatric conditions^140–143^. Moreover, it has been shown that additional diagnosis beyond the primary diagnosis may affect the degree of cortical thickness changes, for example in ADHD with BD and MDD with anxiety^144–146^. As data used in our study were collected from many independent research centers, comorbidities and medication effects could not be fully adjusted. Future studies need to consider controlling for such confounders. Second, we associated the shared disease dimension with features from multiple neural scales derived from independent cohorts, which precludes a direct interpretation of transdiagnostic effects with respect to histological as well as molecular mechanisms in the same subjects. Future studies, likely very challenging to accomplish, that measure multiscale features from the same individuals may set basis for more direct interpretations. Third, the cortical morphological data from the ENIGMA dataset were only available in the Desikan–Killiany parcellation^67^, a macroscopic scheme following sulco-gyral patterns. In addition to not offering a high granularity on cortical arealization, the reliance on folding patterns alone may only provide rather limited sensitivity to contextualize our findings with respect to functional topographies. It would, thus, be relevant to re-evaluate functional gradient association based on functionally-defined parcellations^147,148^, and/or to assess vertex-level feature data in future efforts. Lastly, through the investigation of the mean effect, we observed that the primary motor cortex is least vulnerable to the conditions we observed. However, the low absolute mean effect in sensory/motor regions does not per se indicate that these regions are less important in the understanding of psychiatric conditions. They may have potential effects that have not been detectable with our analyses. For example, prior functional connectivity studies observed that transdiagnostic effects may often be detectable in sensory and motor cortices^149,150^, in addition to heteromodal and paralimbic areas^82,83^.

As a final integrative analysis, we opted for a supervised statistical learning paradigm to predict the shared disease effect from combinations of neuroarchitectural features. This analysis indeed underscored that not a single feature, but rather combinations of microarchitectural and transmitter systems, have the highest utility in predicting the spatial pattern of the transdiagnostic morphological dimension. By, thus, highlighting microstructural and functional aspects of local cortical circuitry, our data-driven findings provide insights into potential determinants of transdiagnostic effects. Overall, our findings emphasize that an increasingly recognized principal gradient that differentiates sensory/motor networks from transmodal cortices in healthy brains^38,40,84^ also describes the main axis of cortex-wide susceptibility to transdiagnostic effects of common mental health conditions. Altogether, the findings may provide a potentially integrative framework for understanding neuropathology in psychiatry, and potentially inform the development of diagnostic and treatment strategies that cut across traditional disease boundaries.

## Methods

### Study dataset

#### ENIGMA data

We analyzed T1-weighted data from people with a diagnosis of (*n* = 12,876) ASD (*n* = 1821), ADHD (*n* = 1815), MDD (*n* = 2695), OCD (*n* = 2274), BD (*n* = 1555), and SCZ (*n* = 2716) and site-matched healthy controls (*n* = 15,670) from 145 independent cohorts participating in prior ENIGMA consortium studies^26–31^. Demographic information is summarized in Supplementary Table 1 and available in a recent cross-condition study^33^. Data from each center were processed using the standard ENIGMA workflow (http://enigma.ini.usc.edu/protocols/imaging-protocols/). Processing was conducted using FreeSurfer^151–153^ that involves magnetic field inhomogeneity correction, non-brain tissue removal, intensity normalization, and tissue segmentation. Estimated white and pial surfaces were inflated to spheres and registered to the *fsaverage* template. Based on the Desikan–Killiany atlas^67^, cortical thickness was measured for 68 gray matter brain regions. For each psychiatric condition, the ENIGMA groups performed multiple linear regression analyses to fit cortical thickness measures with age, age squared, sex, and site information. The meta-analytic profiles of between-group differences between patients and controls were estimated via an inverse variance-weighted random-effects model, which can be obtained from the previous study^33^ (Fig. 1a). If the studies provided multiple effect sizes across children/adolescents/adults, only the effects from the adult sample were used, in order to match the age range across conditions. The positive/negative effects indicate increases/decreases in cortical thickness in patients relative to controls. Individual cohort investigators obtained approval from local institutional ethics boards, and informed consent was obtained from study participants or their guardians.

#### HCP data

To generate microstructural and functional connectivity gradients, we also studied 207 unrelated healthy young adults (60% females, mean age ± SD = 28.73 ± 3.73 years) from the HCP dataset^65^. In the HCP, multimodal imaging data comprising T1- and T2-weighted as well as rs-fMRI were acquired on a Siemens Skyra 3 T at Washington University. The cohort selection is identical to our prior work^32,154^. T1-weighted images were acquired using a magnetization-prepared rapid gradient-echo (MPRAGE) sequence (repetition time (TR) = 2400 ms; echo time (TE) = 2.14 ms; inversion time (TI) = 1000 ms; flip angle = 8°; field of view (FOV) = 224 mm^2^ × 224 mm^2^; voxel size = 0.7 mm isotropic; 256 slices). T2-weighted data were obtained using a T2-SPACE sequence, with the same acquisition parameters as for the T1-weighted data except for TR (3200 ms), TE (565 ms), and flip angle (variable). The rs-fMRI data were collected using a gradient-echo echo-planar imaging sequence (TR = 720 ms; TE = 33.1 ms; flip angle = 52°; FOV = 208 mm^2^ × 180 mm^2^; voxel size = 2 mm isotropic; the number of slices = 72; and 1200 volumes per time series), where participants were instructed to keep their eyes open looking at a fixation cross during the scan. Two sessions (left-to-right and right-to-left phase-encoded directions) of rs-fMRI data were acquired, providing up to four time series per participant. Participant recruitment procedures and informed consent forms, including consent to share de-identified data, were previously approved by the Washington University Institutional Review Board as part of the HCP.

Images underwent minimal preprocessing pipelines using FSL, FreeSurfer, and Workbench as follows^155–157^:

#### T1- and T2-weighted data

Data were corrected for gradient nonlinearity and b0 distortions, and then T1- and T2-weighted data were co-registered using a rigid-body transformation. The bias field was adjusted based on the inverse intensities from the T1- and T2-weighting. The white and pial surfaces were generated^151–153^, and the mid-thickness surface was generated by averaging them. The mid-thickness surface was inflated and the spherical surface was registered to the Conte69 template with 164k vertices^158^ using MSMAll^148^ and downsampled to a 32k vertex mesh.

#### Microstructure data

Myelin-sensitive proxy was estimated based on the ratio of the T1- and T2-weighted contrast^159,160^. We generated 14 equivolumetric surfaces within the cortex and sampled T1w/T2w intensity along these surfaces^38^. A microstructural similarity matrix was constructed by calculating the linear correlation of cortical depth-dependent T1w/T2w intensity profiles between different cortical regions based on the Desikan–Killiany atlas^67^, controlling for the average whole-cortex intensity profile^38^. The matrix was thresholded at zero and log-transformed^38^. A group matrix was constructed by averaging matrices across participants.

#### rs-fMRI data

Data were corrected for distortions and head motion, and registered to the T1-weighted data and subsequently to MNI152 standard space. Magnetic field bias correction, skull removal, and intensity normalization were performed. Noise components attributed to head movement, white matter, cardiac pulsation, arterial, and large vein related contributions were removed using FMRIB’s ICA-based X-noiseifier (ICA-FIX)^161^. Preprocessed time series were mapped to the standard “grayordinate” space using a cortical ribbon-constrained volume-to-surface mapping algorithm. The total mean of the time series of each left-to-right/right-to-left phase-encoded data was subtracted to adjust the discontinuity between the two datasets and then concatenated to form a single time series. A functional connectivity matrix was constructed by calculating the linear time series correlations between Desikan–Killiany parcels^67^, followed by Fisher’s r-to-z transformation^162^. Individual connectivity matrices were averaged to construct a group level connectome.

### Shared effects of cortical thickness differences across conditions

To assess transdiagnostic effects of cortical thickness differences in patients relative to controls, we applied principal component analysis to the concatenated effect size maps across six conditions^163^ (Fig. 1b and Supplementary Fig. 1a). The first principal dimension was determined as the shared disease effect. We summarized the effects according to seven intrinsic functional communities^68^, as well as four cortical hierarchical levels^41^. We additionally calculated the mean effect size across the conditions to intuitively interpret shared disease effect (Supplementary Fig. 1b) and also estimated the principal dimension based on the data sourced from the ENIGMA toolbox (i.e., Cohen’s d; Supplementary Fig. 1c). We compared the shared dimension and the effect size of each condition via linear correlations to assess the degree of contribution of each condition (Supplementary Fig. 2). The significance of the correlation was determined using 1000 nonparametric spin-tests, to account for spatial autocorrelation^164^, and corrected for multiple comparisons using an FDR procedure^165^. To assess robustness, we performed leave-one-condition-out cross-validation. Specifically, we estimated the shared dimension using five conditions by excepting for a single condition, and assessed similarity with the shared disease effect estimated based on the whole six conditions (Supplementary Fig. 3). We calculated the significance of the correlation using 1000 spin-tests and multiple comparisons were corrected using FDR^164,165^. We furthermore obtained the map of cortical expansion and functional reconfiguration^69^ and calculated correlations with three shared disease maps, where the significance was determined using a 1000 spin-test.

### Associations to microstructural and functional connectivity gradients

We evaluated the underlying connectome organizations of the shared disease effects. Based on T1w/T2w and rs-fMRI data obtained from the HCP database^65^, we estimated microstructural and functional gradients, the low dimensional representation of connectome organizations explaining spatial variation in the connectome data^38,40^, using BrainSpace (https://github.com/MICA-MNI/BrainSpace)^166^ (Fig. 1c). An affinity matrix was constructed with a normalized angle kernel from the group averaged connectivity matrix with the top 10% entries for each parcel. The connectome gradients were estimated using diffusion map embedding^167^, which is robust to noise and computationally efficient compared to other nonlinear manifold learning techniques^77,168^. It is controlled by two parameters α and t, where α controls the influence of the density of sampling points on the manifold (α = 0, maximal influence; α = 1, no influence) and t scales eigenvalues of the diffusion operator. The parameters were set as α = 0.5 and t = 0 to retain the global relations between data points in the embedded space, following prior applications^38,40,48,166,169^. We associated the shared effect with these gradients using linear correlation (Fig. 1d), where the significance was assessed using 1000 spin-tests followed by FDR^164,165^.

### Cytoarchitectonic associations with shared disease effects

We aimed to associate the shared dimensions with histology-driven cytoarchitectonic features derived from BigBrain surfaces with 62 cortical areas (https://bigbrain.loris.ca/main.php)^66^. Specifically, BigBrain is a ultra-high resolution, 3D volumetric reconstruction of a postmortem Merker-stained and sliced human brain from a 65-year-old male, with specialized pial and white matter surface reconstructions^66^. The postmortem brain was paraffin-embedded, coronally sliced into 7400 20-μm sections, silver-stained for cell bodies^170^, and digitized. A 3D reconstruction was implemented with a successive coarse-to-fine hierarchical procedure, resulting in a full brain volume. Among 68 regions defined by the Desikan–Killiany atlas^67^, three regions per hemisphere, including banks of the superior temporal sulcus, frontal pole, and temporal pole, were excluded as the BigBrain did not provide data for these regions. We generated 18 equivolumetric cortical surfaces within the cortex (https://github.com/caseypaquola/BigBrainWarp) and sampled the intensity values along these surfaces. Based on the intensity values, we calculated four moment features, including mean, SD, skewness, and kurtosis, as well as externopyramidization (Fig. 2a, b). The mean and SD represent the overall intensity distribution of cytoarchitecture across layers, skewness indicates shifts in intensity values towards supragranular layers (i.e., positive skewness) or flat distribution (i.e., negative skewness), and kurtosis identifies whether the tails of the intensity distribution contain extreme values. Externopyramidization reflects gradual shifts of intensity values from infragranular to supragranular layers defined as follows^75^:

1 $${{{{{{\mathrm{Externopyramidization}}}}}}}=\frac{({{{{{{\mathrm{intensity}}}}}}})}{{{{{{{\mathrm{mean}}}}}}} \, ({{{{{{\mathrm{intensity}}}}}}})}\times \frac{1-{{{{{{\mathrm{thickness}}}}}}}_{{{{{{{\mathrm{supra}}}}}}}}}{{{{{{{\mathrm{thickness}}}}}}}_{{{{{{{\mathrm{total}}}}}}}}}$$

To assess associations with shared disease effects, we calculated linear correlations between cytoarchitectonic features and shared effects (Fig. 2c). The significance of the correlations was assessed using 1,000 spin-tests followed by FDR across different cytoarchitectonic features^164,165^. BigBrain is a 3D model of the human brain with microscopic resolution, enabling us to assess cellular organization in cortical layers^66^. However, it is based a single and significantly older subject compared to our study participants. We thus additionally calculated intracortical profile moments as well as externopyramidization from in vivo myelin-sensitive MRI i.e., the ratio of T1- and T2-weighted contrast^159,160^, obtained from the HCP database^65^. We associated the features with shared disease effect, and the correlations were assessed using 1,000 spin-tests followed by FDR^164,165^.

### Associations between transmitter systems and shared effects

To provide underlying molecular properties of the shared effects in neuroanatomical disruptions across different psychiatric conditions, we associated the shared dimensions with ten different neurotransmitter maps of healthy controls provided by prior independent PET/SPECT studies^54–59^, which contain neurotransmitters of FDOPA, GABAa, transporters of DAT, NAT, SERT, and receptors of D1, D2, 5-HT1a, 5-HT1b, and 5-HT2a (https://github.com/juryxy/JuSpace)^53^ (Fig. 3a). All PET maps were linearly rescaled to have intensity values between 0 and 100^53^. After mapping the neurotransmitter maps onto the Desikan–Killiany atlas^67^, we calculated linear correlations between the shared effects and each neurotransmitter map (Fig. 3b), and assessed the significance using 1000 spin-tests followed by FDR to adjust for multiple comparisons across ten different maps^164,165^.

### Associations between multiscale features and other shared dimensions

We furthermore associated the second shared dimensions (Supplementary Fig. 1a) with microstructural and functional connectivity gradients, cytoarchitectural moments, and neurotransmitter system distributions to assess convergence or divergence across the shared dimensions (Supplementary Table 2). We also estimated a shared disease dimension based on surface area, souring Cohen’s d maps from the ENIGMA toolbox^32^. Here, we applied principal component analysis to the concatenated effect size maps of surface area across five conditions, as ASD was not available (Supplementary Fig. 5 and Supplementary Table 3).

### Prediction of shared effects using multiscale features

We associated multiscale features and shared effects using supervised machine learning to incorporate our findings (Fig. 4). Specifically, we aimed to predict the shared disease effects using concatenated multiscale features of microstructural and functional gradients, cytoarchitectonic (i.e., mean, SD, skewness, kurtosis, and externopyramidization), and transmitter maps (i.e., D1, D2, 5-HT1a, 5-HT1b, 5-HT2a, FDOPA, GABAa, DAT, NAT, and SERT). We used five-fold nested cross-validation^78–80^ with LASSO regression^76^. Nested cross-validation split the dataset into training (4/5) and test (1/5) partitions, and each training partition was further split into inner training and testing folds using another five-fold cross-validation. The model with the best performance (lowest MAE) across the inner folds was applied to the test partition of the outer fold. Among the multiscale features, we selected performant features using LASSO regularization, and the effect size was predicted using linear regression with the selected features. The procedure was repeated 100 times with different training and test partitions. Prediction accuracy was evaluated with linear correlations between the actual and predicted effect size and the MAE, with their 95% confidence interval. Permutation-based correlations across 1000 tests were conducted by randomly shuffling cortical regions to verify whether the prediction performance exceeded chance levels. We also performed the prediction analysis using the effect size of each condition (Supplementary Fig. 6).

### Statistics and reproducibility

The between-group differences in cortical thickness of each psychiatric condition were assessed using inverse variance-weighted random-effects models^33^, and their shared disease effects were estimated via principal component analysis. We assessed associations between the shared disease dimension and microstructural and functional connectivity gradients, cytoarchitectonic features calculated from the BigBrain^66^, and neurotransmitter maps obtained from independent PET/SPECT studies^54–59^ based on linear correlations with 1000 spin-tests followed by FDR^164,165^. We opted for supervised machine learning to associate multiscale features and shared effects based on five-fold nested cross-validation^78–80^ with LASSO regression^76^.

### Reporting summary

Further information on research design is available in the Nature Research Reporting Summary linked to this article.

## Supplementary information

Supplementary Information

Description of Additional Supplementary Files

Supplementary Data 1

Reporting Summary

## Data Availability

Disorder-related effect size measures analyzed in this project are openly available via https://enigma-toolbox.readthedocs.io and 10.1001/jamapsychiatry.2020.2694. Raw imaging data that support these findings are not publicly available in a repository as they contain information that could compromise the privacy of research participants. Although there are data sharing restrictions imposed by (i) ethical review boards of the participating sites, and consent documents; (ii) national and trans-national data sharing law, such as GDPR; and (iii) institutional processes, some of which require a signed MTA for limited and predefined data use, we welcome sharing data with researchers, requiring only that they submit an analysis plan for a secondary project to the leading team of the Working Group (http://enigma.ini.usc.edu). Once this analysis plan is approved, access to the relevant data will be provided contingent on data availability and local PI approval and compliance with all supervening regulations. If applicable, distribution of analysis protocols to sites will be facilitated. Source data (Supplementary Data 1) are provided with this paper.

## Code availability

Codes for multiscale neural feature calculation and statistical analyses are provided at BrainSpace (10.1038/s42003-020-0794-7; https://github.com/MICA-MNI/BrainSpace) and ENIGMA toolbox (10.1038/s41592-021-01186-4; https://github.com/MICA-MNI/ENIGMA). Tutorials to carry out similar analyses are provided on https://enigma-toolbox.readthedocs.io/en/latest/.
