# Supplementary material for: Multiscale neural gradients reflect transdiagnostic effects of major psychiatric conditions on cortical morphology
Source: Commun Biol. 2022 Sep 27;5:1024. doi: 10.1038/s42003-022-03963-z (PMC9515219; doi:10.1038/s42003-022-03963-z)
Supplement: Supplementary file 1 — Supplementary Information [file 42003_2022_3963_MOESM1_ESM.pdf]

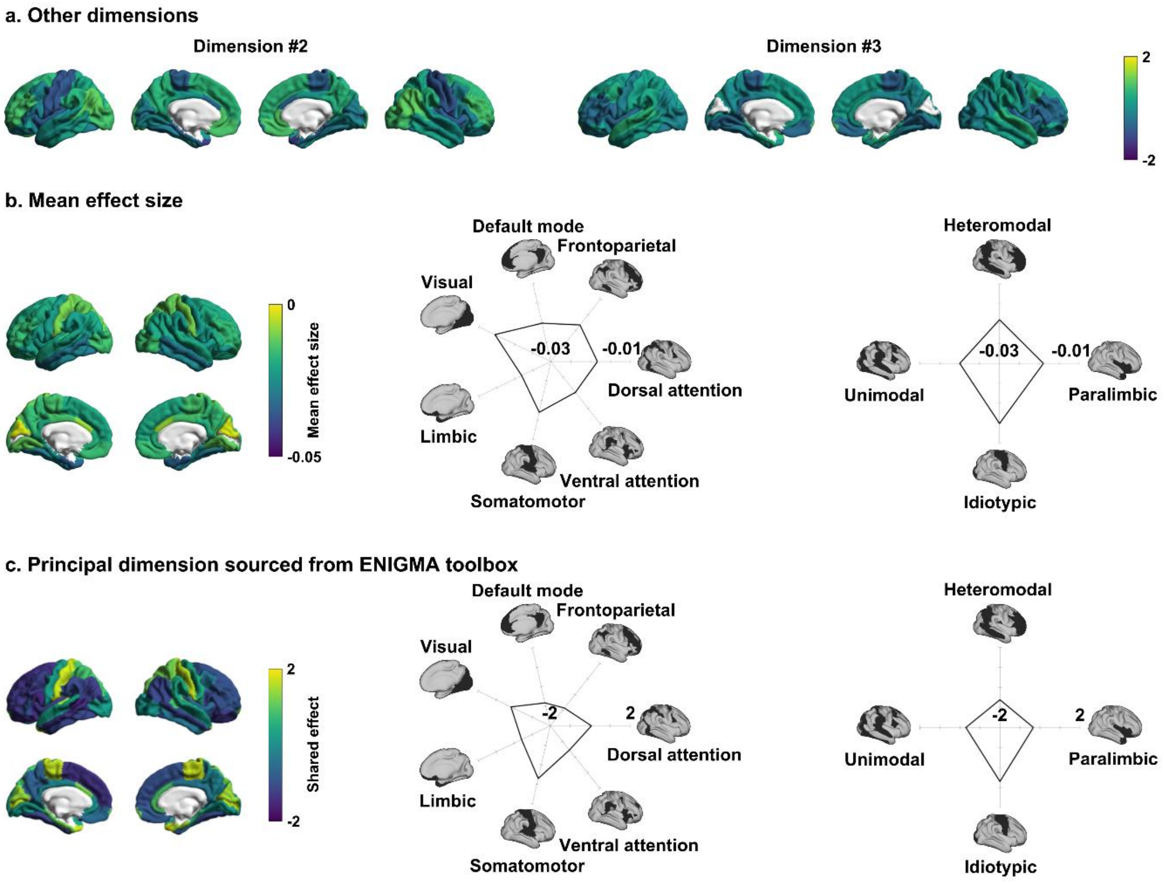

Supplementary Figure 1 | Shared disease effect. (A) The second and third dimensions of the shared disease effects, explaining 17.5% and 9.2% of variance, respectively. **(B)** Mean effect size of cortical thickness alterations across conditions. **(C)** Principal dimension based on the effect size maps (Cohen's d) sourced from the ENIGMA toolbox. The effects were stratified according to functional communities and cortical hierarchy levels.

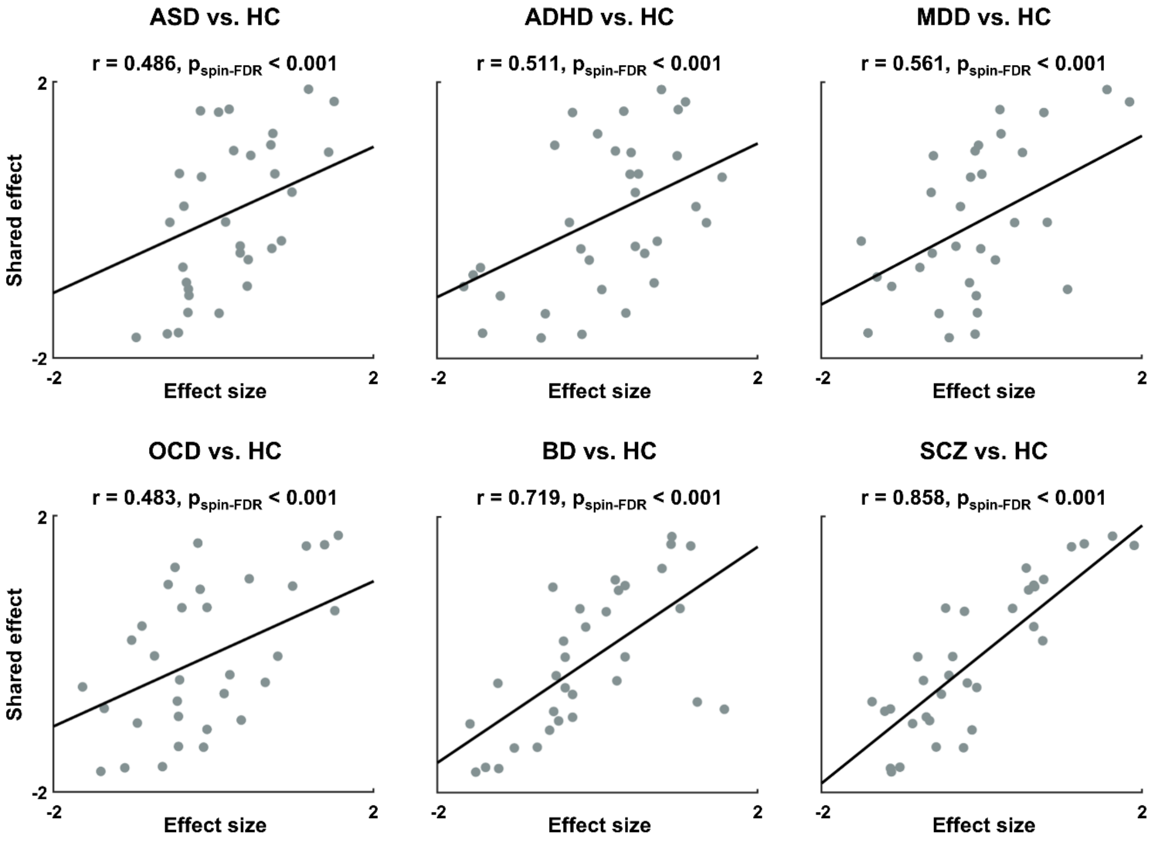

Supplementary Figure 2 | Linear correlations between the shared disease effect and cortical thickness alterations in each psychiatric condition. *Abbreviations:* ASD, autism spectrum disorder; ADHD, attention deficit hyperactivity disorder; MDD, major depressive disorder; OCD, obsessive-compulsive disorder; BD, bipolar disorder; SCZ, schizophrenia; HC, healthy controls; spin-FDR, spin-test followed by false discovery rate.

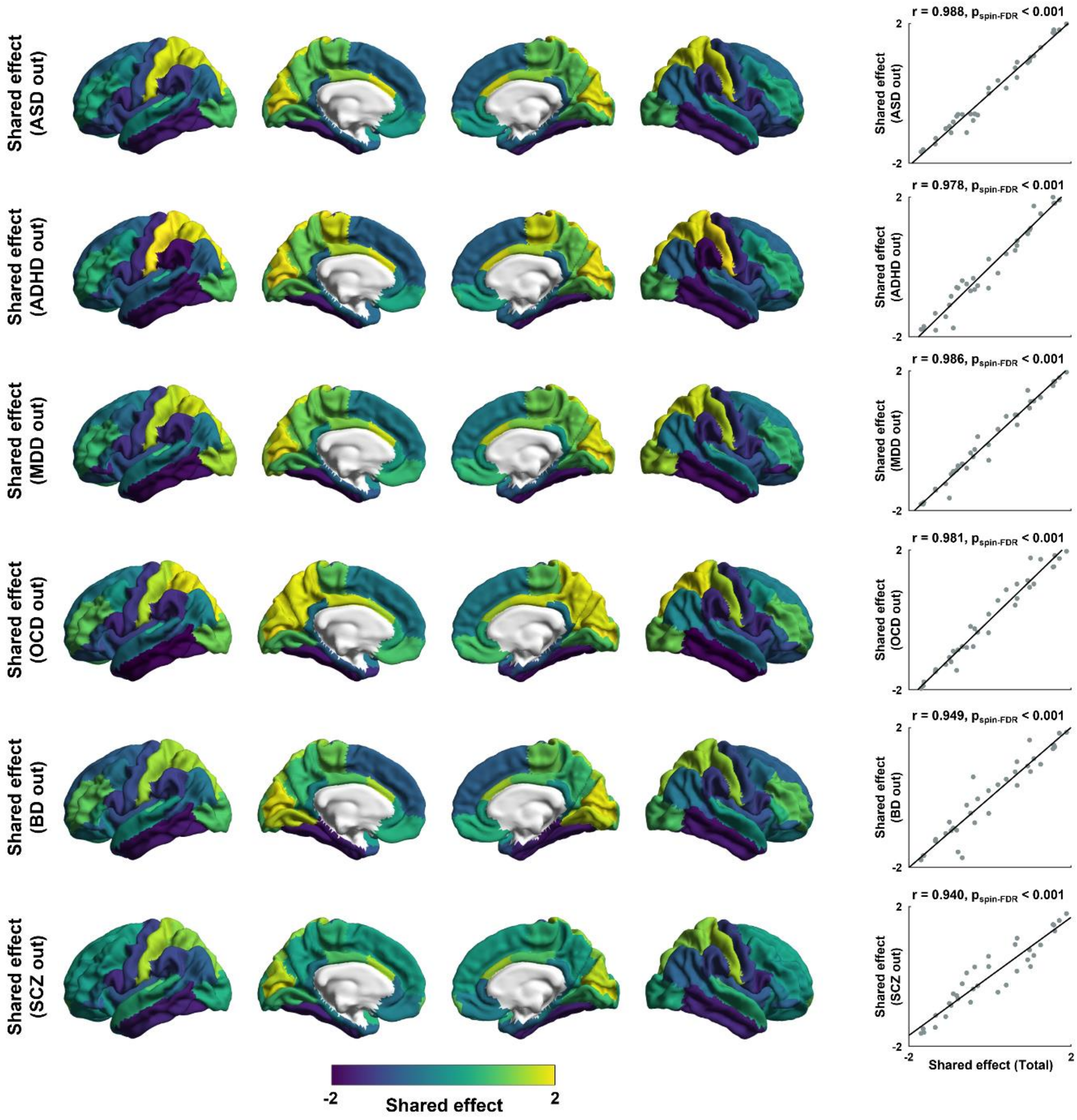

Supplementary Figure 3 | Shared disease effects with leave-one-condition-out cross-validation. The shared dimensions estimated based on all conditions without a single condition are reported on brain surfaces. Linear correlations between the shared effect based on all conditions (see *Fig. 1B*) and that based on five conditions are shown in the scatter plots. *Abbreviations:* ASD, autism spectrum disorder; ADHD, attention deficit hyperactivity disorder; MDD, major depressive disorder; OCD, obsessive-compulsive disorder; BD, bipolar disorder; SCZ, schizophrenia; HC, healthy controls; spin-FDR, spin-test followed by false discovery rate.

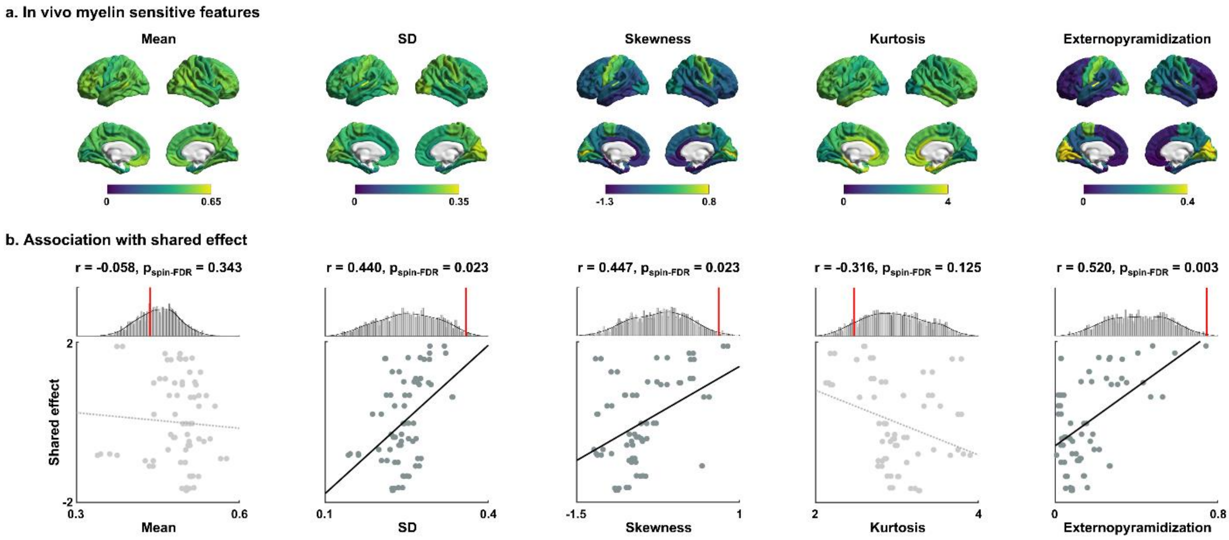

Supplementary Figure 4 | Associations between the shared disease effect and *in vivo* myelin sensitive imaging based on T1w/T2w ratio obtained from the HCP database. (A) Moment features as well as externopyramidization calculated from *in vivo* myelin sensitive imaging. **(B)** Spatial correlations of the features with the shared disease effect. For details, see *Fig. 2*. *Abbreviations:* SD, standard deviation; spin-FDR, spin-test followed by false discovery rate.

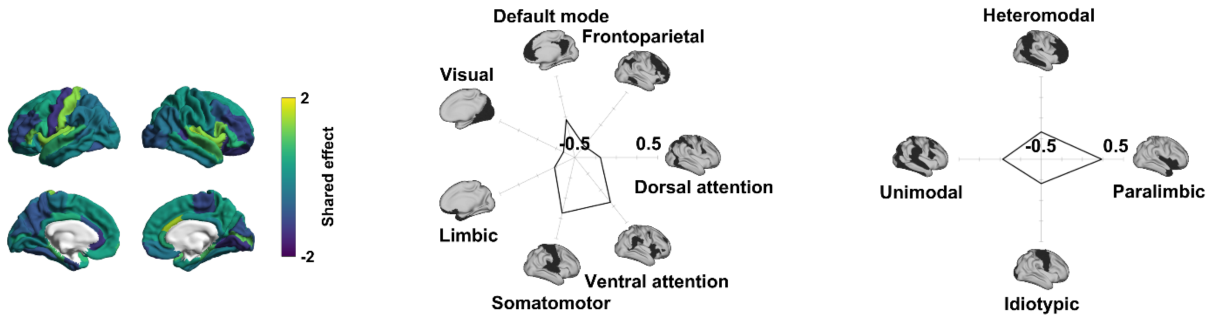

Supplementary Figure 5 | Shared disease effects of surface area. The shared effect was identified using the concatenated effect size maps of surface area, and stratified according to functional communities and cortical hierarchy levels.

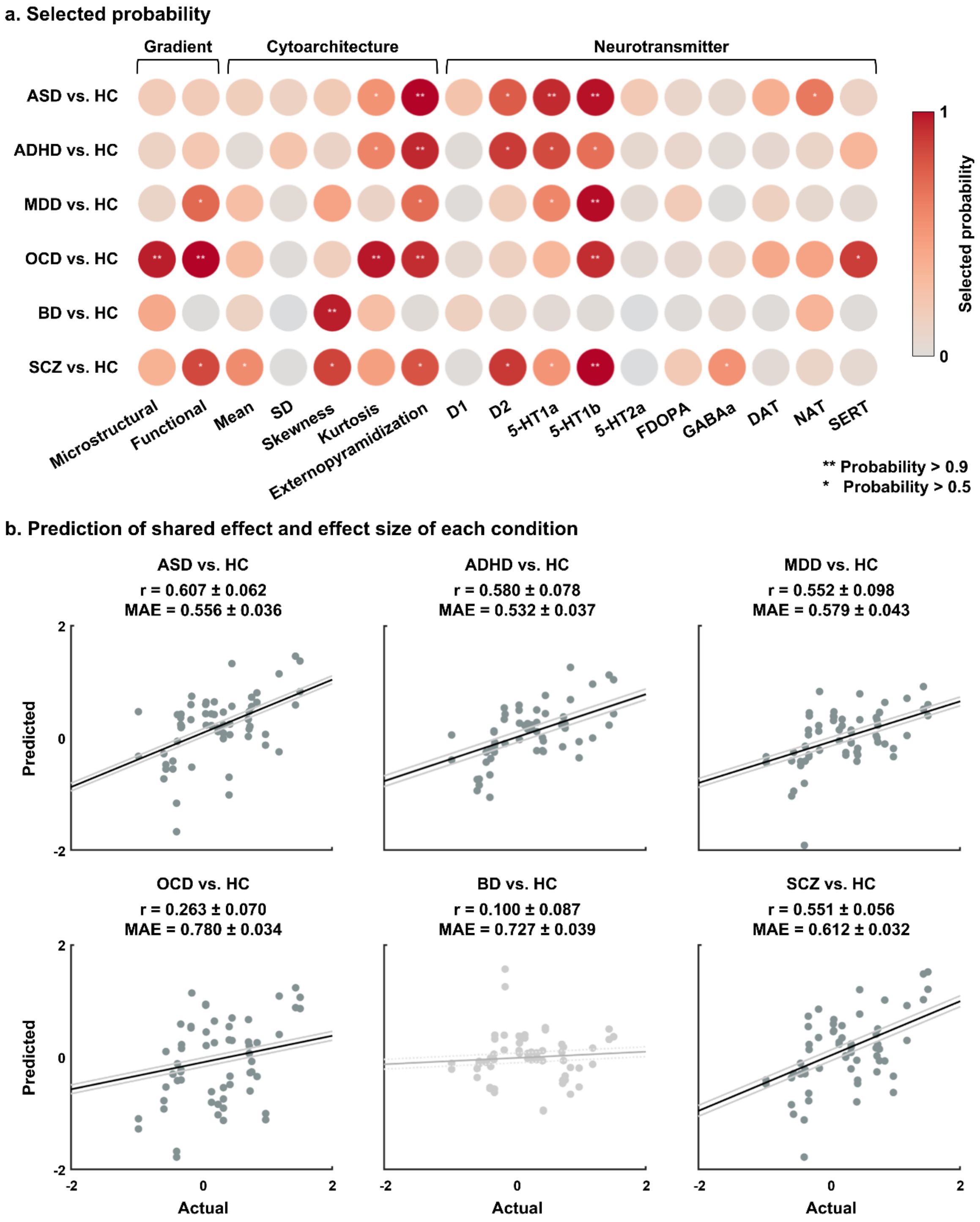

Supplementary Figure 6 | Association between the effect size of each psychiatric condition and multiscale features. (A) Probability of the selected features for each psychiatric condition. **(B)** Linear correlations between actual and predicted values of the effects are shown using scatter plots. Black lines indicate mean correlation and gray lines represent the 95% confidence interval for 100 iterations with different training/test datasets. For details, see *Fig. 4*. *Abbreviations:* ASD, autism spectrum disorder; ADHD, attention deficit hyperactivity disorder; MDD, major depressive disorder; OCD, obsessive-compulsive disorder; BD, bipolar disorder; SCZ, schizophrenia; HC, healthy controls; SD, standard deviation; FDOPA, 18F fluorodopa; DAT, dopamine transporter; NAT, noradrenaline transporter; SERT, serotonin transporter; MAE, mean absolute error.

Supplementary Table 1 | Demographic information of studied participants.

Condition	Number	Mean (SD; range) age (years)	Sex (male:female)
ASD/controls	1821/1823	15.6 (6.7; 2–64)	2941:703 (19% female)
ADHD/controls	1815/1602	21.1 (5.4; 4–74)	2244:1172 (34% female)
MDD/controls	2695/3627	40.9 (10.9; 8–89)	2665:3657 (58% female)
OCD/controls	2274/2013	27.2 (8.0; 5–65)	2166:2121 (49% female)
BD/controls	1555/3423	35.1 (12.0; 8–86)	2142:2836 (57% female)
SCZ/controls	2716/3272	33.9 (10.7; 7–87)	3479:2509 (42% female)

Detailed information available in eTable 1 and eTable 2 of Patel et al., 2021. *Abbreviations:* SD, standard deviation; ASD, autism spectrum disorder; ADHD, attention deficit hyperactivity disorder; MDD, major depressive disorder; OCD, obsessive-compulsive disorder; BD, bipolar disorder; SCZ, schizophrenia.

Supplementary Table 2 | Associations between multilevel features and other principal dimensions.

Features		1st dimension		2nd dimension	
		r	$p_{spin\text{-}FDR}$	r	$p_{spin\text{-}FDR}$
**Gradient**	Microstructure	-0.400	0.042*	0.195	0.078
	Function	-0.247	0.090	0.364	0.003*
**Cytoarchitecture**	Mean	0.200	0.167	0.107	0.265
	SD	0.262	0.167	0.143	0.263
	Skewness	0.400	0.015*	-0.132	0.263
	Kurtosis	0.132	0.167	0.140	0.263
	Externo-pyramidization	0.472	0.015*	0.091	0.265
**Neurotransmitter**	DAT	-0.240	0.041*	-0.124	0.193
	NAT	0.217	0.073	-0.144	0.172
	SERT	-0.042	0.375	-0.191	0.099
	D1	-0.206	0.073	0.176	0.142
	D2	0.280	0.035*	0.304	0.013*
	5-HT1a	-0.307	0.033*	-0.094	0.251
	5-HT1b	0.349	0.025*	0.321	0.017*
	5-HT2a	0.091	0.251	0.350	0.013*
	FDOPA	-0.175	0.103	-0.065	0.296
	GABAa	0.100	0.243	0.272	0.030

Significant associations are reported with asterisks. *Abbreviation:* SD, standard deviation; FDOPA, 18F fluorodopa; DAT, dopamine transporter; NAT, noradrenaline transporter; SERT, serotonin transporter; spin-FDR, spin-test followed by false discovery rate.

Supplementary Table 3 | Associations between multiscale features and shared disease dimension of surface area.

Features		r	$p_{spin\text{-}FDR}$
**Gradient**	Microstructure	-0.043	0.393
	Function	-0.022	0.430
**Cytoarchitecture**	Mean	0.011	0.453
	SD	-0.115	0.246
	Skewness	0.013	0.474
	Kurtosis	0.169	0.094
	Externopyramidization	-0.031	0.430
**Neurotransmitter**	DAT	0.158	0.159
	NAT	0.083	0.267
	SERT	0.274	0.029*
	D1	-0.084	0.277
	D2	-0.223	0.079
	5-HT1a	-0.099	0.248
	5-HT1b	-0.182	0.122
	5-HT2a	-0.297	0.011*
	FDOPA	-0.141	0.148
	GABAa	-0.325	0.005*

Significant associations are reported with asterisks. *Abbreviations:* SD, standard deviation; FDOPA, 18F fluorodopa; DAT, dopamine transporter; NAT, noradrenaline transporter; SERT, serotonin transporter; spin-FDR, spin-test followed by false discovery rate.
