# Supplementary material for: Multiscale neural gradients reflect transdiagnostic effects of major psychiatric conditions on cortical morphology
Source: Commun Biol. 2022 Sep 27;5:1024. doi: 10.1038/s42003-022-03963-z (PMC9515219; doi:10.1038/s42003-022-03963-z)
Supplement: Supplementary file 4 — Reporting Summary [file 42003_2022_3963_MOESM4_ESM.pdf]

nature portfolio

Corresponding author(s): Bo-yong Park
Boris C. Bernhardt
COMMSBIO-22-1789-T

Last updated by author(s): Aug 17, 2022

Reporting Summary

Nature Portfolio wishes to improve the reproducibility of the work that we publish. This form provides structure for consistency and transparency in reporting. For further information on Nature Portfolio policies, see our Editorial Policies and the Editorial Policy Checklist.

Statistics

For all statistical analyses, confirm that the following items are present in the figure legend, table legend, main text, or Methods section.

n/a	Confirmed	
☐	☒	The exact sample size (*n*) for each experimental group/condition, given as a discrete number and unit of measurement
☒	☐	A statement on whether measurements were taken from distinct samples or whether the same sample was measured repeatedly
☐	☒	The statistical test(s) used AND whether they are one- or two-sided<br>*Only common tests should be described solely by name; describe more complex techniques in the Methods section.*
☐	☒	A description of all covariates tested
☐	☒	A description of any assumptions or corrections, such as tests of normality and adjustment for multiple comparisons
☐	☒	A full description of the statistical parameters including central tendency (e.g. means) or other basic estimates (e.g. regression coefficient) AND variation (e.g. standard deviation) or associated estimates of uncertainty (e.g. confidence intervals)
☐	☒	For null hypothesis testing, the test statistic (e.g. *F*, *t*, *r*) with confidence intervals, effect sizes, degrees of freedom and *P* value noted<br>*Give P values as exact values whenever suitable.*
☒	☐	For Bayesian analysis, information on the choice of priors and Markov chain Monte Carlo settings
☒	☐	For hierarchical and complex designs, identification of the appropriate level for tests and full reporting of outcomes
☐	☒	Estimates of effect sizes (e.g. Cohen's *d*, Pearson's *r*), indicating how they were calculated

Our web collection on statistics for biologists contains articles on many of the points above.

Software and code

Policy information about availability of computer code

Data collection	We obtained case-control maps of cortical thickness differences in patients relative to controls, resulting from several ENIGMA working groups aggregated by a previous study that included a total of 28,546 participants across 145 independent cohorts (1,821 ASD, 1,815 ADHD, 2,695 MDD, 2,274 OCD, 1,555 BD, 2,716 SCZ; 15,670 site-matched controls).
Data analysis	BrainSpace (https://github.com/MICA-MNI/BrainSpace), ENIGMA toolbox (https://enigma-toolbox.readthedocs.io)

For manuscripts utilizing custom algorithms or software that are central to the research but not yet described in published literature, software must be made available to editors and reviewers. We strongly encourage code deposition in a community repository (e.g. GitHub). See the Nature Portfolio guidelines for submitting code & software for further information.

Data

Policy information about availability of data

All manuscripts must include a data availability statement. This statement should provide the following information, where applicable:

- Accession codes, unique identifiers, or web links for publicly available datasets
- A description of any restrictions on data availability
- For clinical datasets or third party data, please ensure that the statement adheres to our policy

Disorder related effect size measures analyzed in this project are openly available via https://enigma-toolbox.readthedocs.io and doi: 10.1001/jamapsychiatry.2020.2694.

Raw imaging data that support these findings are not publicly available in a repository as they contain information that could compromise the privacy of research participants. Although there are data sharing restrictions imposed by (i) ethical review boards of the participating sites, and consent documents; (ii) national and trans-national data sharing law, such as GDPR; and (iii) institutional processes, some of which require a signed MTA for limited and predefined data use, we welcome sharing data with researchers, requiring only that they submit an analysis plan for a secondary project to the leading team of the Working Group (http://enigma.ini.usc.edu). Once this analysis plan is approved, access to the relevant data will be provided contingent on data availability and local PI approval and compliance with all supervening regulations. If applicable, distribution of analysis protocols to sites will be facilitated.

Human research participants

Policy information about studies involving human research participants and Sex and Gender in Research.

Reporting on sex and gender

Condition	Sex (male:female)
ASD/controls	2941:703 (19% female)
ADHD/controls	2244:1172 (34% female)
MDD/controls	2665:3657 (58% female)
OCD/controls	2166:2121 (49% female)
BD/controls	2142:2836 (57% female)
SCZ/controls	3479:2509 (42% female)

Population characteristics

Condition	Number	Mean (SD; range) age (years)	Sex (male:female)
ASD/controls	1821/1823	15.6 (6.7; 2–64)	2941:703 (19% female)
ADHD/controls	1815/1602	21.1 (5.4; 4–74)	2244:1172 (34% female)
MDD/controls	2695/3627	40.9 (10.9; 8–89)	2665:3657 (58% female)
OCD/controls	2274/2013	27.2 (8.0; 5–65)	2166:2121 (49% female)
BD/controls	1555/3423	35.1 (12.0; 8–86)	2142:2836 (57% female)
SCZ/controls	2716/3272	33.9 (10.7; 7–87)	3479:2509 (42% female)

Recruitment

People with a diagnosis of (n = 12,876) ASD (n = 1,821), ADHD (n = 1,815), MDD (n = 2,695), OCD (n = 2,274), BD (n = 1,555), and SCZ (n = 2,716) and site matched healthy controls (n = 15,670) were obtained from 145 independent cohorts participating in prior ENIGMA consortium studies

Ethics oversight

Individual cohort investigators obtained approval from local institutional ethics boards, and informed consent was obtained from study participants or their guardians.

Note that full information on the approval of the study protocol must also be provided in the manuscript.

Field-specific reporting

Please select the one below that is the best fit for your research. If you are not sure, read the appropriate sections before making your selection.

☒ Life sciences ☐ Behavioural & social sciences ☐ Ecological, evolutionary & environmental sciences

For a reference copy of the document with all sections, see nature.com/documents/nr-reporting-summary-flat.pdf

Life sciences study design

All studies must disclose on these points even when the disclosure is negative.

Sample size

28,546 participants across 145 independent cohorts (1,821 ASD, 1,815 ADHD, 2,695 MDD, 2,274 OCD, 1,555 BD, 2,716 SCZ; 15,670 site-matched controls)

Data exclusions

N/A

Replication

N/A

Randomization

We calculated significance of the correlation using 1,000 spin-tests, and multiple comparisons were corrected using FDR.

Blinding

N/A

Reporting for specific materials, systems and methods

We require information from authors about some types of materials, experimental systems and methods used in many studies. Here, indicate whether each material, system or method listed is relevant to your study. If you are not sure if a list item applies to your research, read the appropriate section before selecting a response.

Materials & experimental systems

n/a	Involved in the study
☒	☐ Antibodies
☒	☐ Eukaryotic cell lines
☒	☐ Palaeontology and archaeology
☒	☐ Animals and other organisms
☒	☐ Clinical data
☒	☐ Dual use research of concern

Methods

n/a	Involved in the study
☒	☐ ChIP-seq
☒	☐ Flow cytometry
☐	☒ MRI-based neuroimaging

Magnetic resonance imaging

Experimental design

Design type: T1-weighted

Design specifications: N/A

Behavioral performance measures: N/A

Acquisition

Imaging type(s): T1-weighted

Field strength: 3T

Sequence & imaging parameters: Variable across sites. Details are at: https://jamanetwork.com/journals/jamapsychiatry/fullarticle/2769908

Area of acquisition: Whole brain

Diffusion MRI: ☐ Used ☒ Not used

Preprocessing

Preprocessing software: FreeSurfer

Normalization: Estimated white and pial surfaces were inflated to spheres and registered to the fsaverage template.

Normalization template: fsaverage

Noise and artifact removal: Magnetic field inhomogeneity correction, non-brain tissue removal, intensity normalization, and tissue segmentation

Volume censoring: N/A

Statistical modeling & inference

Model type and settings: ENIGMA groups performed multiple linear regression analyses to fit cortical thickness measures with age, age squared, sex, and site information. The meta-analytic profiles of between-group differences between patients and controls were estimated via an inverse variance-weighted random-effects model

Effect(s) tested: Meta-analytic profiles of cortical thickness differences (unit in mm).

Specify type of analysis: ☐ Whole brain ☐ ROI-based ☒ Both

Anatomical location(s): Desikan-Killiany

Statistic type for inference (See Eklund et al. 2016): ROI-wise

Correction: 1,000 spin-tests and FDR.

Models & analysis

n/a	Involved in the study
☒	☐ Functional and/or effective connectivity
☒	☐ Graph analysis
☒	☐ Multivariate modeling or predictive analysis
